# The method for quantified analysis and pattern visualization for eye blinking using high-frame-rate video

**DOI:** 10.1007/s13534-025-00522-3

**Published:** 2025-11-11

**Authors:** Woon-Hee Lee, Jongmo Seo, Jeong-Min Hwang

**Affiliations:** 1 Department of Electrical and Computer Engineering, Seoul National University, 08826 Seoul, Republic of Korea; 2 Department of Ophthalmology, Seoul National University, 03080 Seoul, Republic of Korea; 3 Department of Ophthalmology, Seoul National University Bundang Hospital, 13620 Seongnam, Republic of Korea

**Keywords:** Eye blinking, Blinking pattern, Blinking visualization, Quantified analysis

## Abstract

This study proposes a visualization and analysis method for eye blinking pattern using high-frame-rate videos. The high-frame-rate video clips for visualization are taken using a camera without additional equipment. The partial video clips of eye blinking except for eyelid flutters and microsleeps are extracted from the entire video clip. The changes in shapes and positions of the upper eyelid during the eye blinking sequences are evaluated, and each eye blinking is visualized as a single image. The various parameters regarding eye blinking are calculated to analyze blinking patterns. The single eye blinking sequence is divided into phases to analyze and classify eye blinking patterns in more detail. In this experiment conducted on 80 volunteers, the proposed method was able to quantitatively analyze eyelid movements, and various parameters related to eye blinking were calculated. Additionally, different types of eye blinking patterns were visualized as graph images, and incomplete eye blinking and consecutive eye blinking were defined and detected. The proposed method can overcome the spatial and situational limitations of conventional bio-signal analysis methods, as it allows non-contact measurement in ordinary environments. In addition, since quantitative eye blink data obtained from high-frame-rate video contain more information than data obtained from bio-signals, it is expected that analysis methods using videos can be easily applied to a wider range of fields.

## Introduction

Humans constantly accumulate information through their sense organs during daily activities, and the most of that information is obtained through visual system. The rapid increase in various media channels is greatly expanding the amount of visual information. In addition, the restrictions of the viewing environment are gradually disappearing because of the development of communication technology and the popularization of mobile devices. Previously, these devices were mainly used in indoor environments with illumination, but those are now also used outdoors or in dark places without any illumination.

These changes are expected to provide an improved visual experience and a more convenient life, but at the same time, they are reported to cause various symptoms such as dryness, foreign body sensation, visual fatigue, and blurred vision through decreased blinking [1, 2]. Accordingly, the number of patients complaining of various ophthalmic diseases is increasing, and interest in these diseases is increasing day by day. Although various methods have been developed to monitor, diagnose, and analyze the ocular condition, these symptoms are mainly subjective and therefore difficult to objectively evaluate.

The most widely used methods for diagnosing ocular conditions are subject-dependent assessment methods, which estimate a patient's ocular condition based on their responses to various questionnaires with scores or grades. Representative examples include visual acuity tests, McMonnies questionnaire for screening tool to diagnose the presence or absence of dry eye disease [3], and other questionnaires for assessing visual function and quality of life such as the Activities of Daily Vision Scale (ADVS) [4], Visual Function Index (VF-14) [5], and low vision quality-of-life questionnaire (LVQOL) [6]. However, these research methods that rely on patients’ answers often yields unsatisfactory results and low reproducibility.

For the objective evaluation of the condition of the eye excluding the subject’s subjectivity and intention, various medical devices are used [7, 8]. These medical devices can be used to precisely and accurately analyze the objective ocular condition. However, such medical devices are expensive and require specialized systems, thus their uses are limited to hospitals or research facilities. In addition, since most examinations require both eyes to make contact with the examination equipment, it is impossible to analyze ocular conditions continuously for a long period of time.

In order to overcome these limitations of the previous evaluation methods, numerous studies have been attempted to analyze to use eye blinking as a surrogate indicator of various eye conditions [9–13]. Eye blinking is a kind of reflex action that occurs continuously during human activity, and can be easily noticed despite its short duration about 100 to 400 ms [14]. It is also known that the eye blinking varies depending on physical conditions, pain, diseases, or various other environmental changes. For these reasons, it has been considered that the eye blinking can be used as an indicator of human condition.

The most typical method to analyze eye blinking is to measure bio-signals such as electro-oculograms (EOG) using a sampling frequency of around 100–200 Hz and investigate the changes in the shape of the bio-signal, which allows for objective and continuous analysis [10, 15]. These studies mainly analyzed the frequency of eye blinking and the duration of each blinking.In addition, some studies analyzed the duration of eyelid closure and opening by dividing the sequence of eye blinking [9]. 

However, methods based on bio-signals still have certain limitations. Studies on eye blinking require long-term analysis, as the tendency and deviation of eye blinking vary from person to person. The limitation of attaching wired electrodes to the subject’s face to measure bio-signals makes long-term or continuous monitoring difficult, so the analysis is only possible for a certain period of time (within a few minutes).

Additionally, the signal can be affected by sudden eye movements or irregular contractions of facial muscles, which makes it difficult to analyze only specific components or to set up the test protocols [16]. Furthermore, since eyelid movements are extremely difficult to quantify using biosignal analysis, it is impossible to analyze blinking in detail or determine whether the eyelids fully close.

To overcome the limitations of eye blink analysis methods using bio-signals, research on eye blink analysis methods using video has been conducted[13]. Video-based eye blink analysis has the advantages of being non-contact, continuous, and quantitative. However, since the eye blinking durations are short (typically around 100–400 ms), the standard video rate of 30 frames per second (fps) is not sufficient to provide the accurate and diverse information regarding eye blinking that bio-signals can provide. Additionally, the difference in the position of the eyelid between two frames may be too large to precisely analyze changes during the blinking [17]. 

In order to utilize eye blinking as a surrogate indicator of various human conditions, it is necessary to develop a novel method of eye blink analysis that overcomes the aforementioned limitations. The method should be non-contact and quantitative, and capable of continuous measurement and monitoring over long periods of time.

In this study, we propose an analysis and visualization method for eye blinking using a high-speed vision sensor to satisfy these conditions. The proposed method can analyze and quantify eyelid movement from high-frame-rate videos, calculate parameters related to blinking, and divide the blinking sequences into phases based on the quantified difference in the eyelid position to analyze and classify eye blinking patterns in more detail. Through this, we aim to simultaneously reap the benefits of both bio-signal analysis with high-sampling-rate and image analysis that enables quantitative evaluation. Furthermore, we visualize each eye blinking as a single image to make it easier to recognize the characteristics and patterns of eye blinking at a glance.

## Method

The proposed analysis method not only calculates various parameters that have been measured in previous studies analyzing eye blinks using bio-signals, but also attempts to quantitatively analyze eyelid movements during blinkings which were difficult to measure using bio-signals.

One approach to locate the eyes and eyelids with high accuracy is to use pattern recognition or deep learning methods, but these methods require a lot of computational time. Recently, studies have been published analyzing eye blinking in 30 fps videos using deep learning [18]. Nevertheless, it is still challenging to process high-frame-rate videos exceeding 200 fps using deep learning, so the eye blinking datasets used in research are generally short videos captured at 30 fps [19]. This situation makes long-term continuous analysis difficult and limits the expansion of application areas.

In this study, we proposed an analysis method suitable for high-frame-rate video analysis that quantitatively evaluates eyelid movements with significantly fewer computations than deep learning approaches.

This experiment was performed with approval and supervision by the institutional review board (IRB) of Seoul National University Hospital (IRB approval number: 1810-112-982), and the subject gave informed consent to their inclusion in the study as required.

### High-frame-rate videos for eye blink analysis

For precise eye blink analysis, the faster the frame rate of the video, the better. However, an excessively fast frame rate acquiring overly more information than necessary requires a lot of computation time for analysis. In the present study, 240 fps videos were mainly used, and this frame rate was sufficient for eye blink analysis because it was similar to the sampling frequency mainly used in EOG analysis [10]. Also, the frame rate was twice the illumination flickering frequency and four times the power frequency at the place where the experiment was conducted. By using a frequency that was a multiple of the illumination flicker frequency, the flicker effect can be easily removed, making image processing and analysis easier.

The videos used for eye blink analysis were acquired using a mobile phone (SM-G965X S9+, Samsung Electronics, Korea) and a low-cost high-frame-rate camera (EX-ZR200, Casio, Japan). Both cameras are able to acquire 240 fps videos for an extended period, but their resolutions differ. The resolution of mobile phone was full HD (1920 × 1080 pixels), and the resolution of camera was 512 × 384 pixels.

In this experiment, additional equipment that could affect eye blinking were excluded as far as possible. Experimental equipment setup for high-frame-rate videos consists simply of a camera and a monitor, and the camera is mounted above the monitor. While an examinee views a video clip on the monitor, the camera captures video of the examinee’s face and the surrounding area. This experimental setup, consisting of only simple equipment, can be easily applied to a variety of environments, such as driving, working on a computer, or watching TV.

### Extraction of eye blinking from the video

The high-frame-rate video clips include a lot of information, so the process of extracting eye blinking sequence from the entire video clip should be preceded for efficient analysis. To extract the eye blink sequence, the color video frames were first converted into gray-scale images. The average pixel intensity calculated from the grayscale images varies continuously during blinking due to the color differences among the skin, eyeball, and pupil. This intensity profile exhibits a periodic change corresponding to each eye blink.

**Fig. 1:**
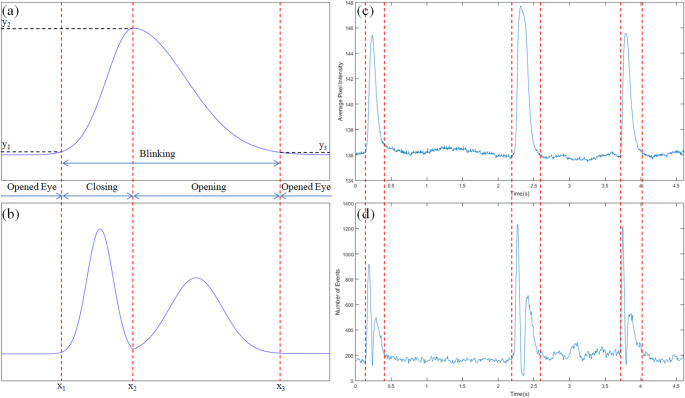
The ideal graph changes during the eye blinking and the result of extracting eye blinking sequences. **a** The ideal signal of gray-scale intensity graph during the eye blinking, **b** The ideal signal of the number of dynamic vision events during the eye blinking, **c** The gray-scale intensity graph during eye blinking in real video clip and extraction results, **d** The number of dynamic vision events graph during eye blinking in real video clip and extraction results

Event signals were additionally generated from gray-scale images. In order to simulate the event signals, each pixel stored a reference brightness level, and continuously compared it to the current brightness level. If the difference in brightness exceeded a threshold, that pixel reset its reference level and generated an event. The amount of dynamic vision events changes twice for each eye blinking (Fig. 1b). The first change in the signal of dynamic vision events represents the eye closing process and the other represents the eye opening process. In general, eye closing movements are more intense and shorter than eye opening movements, so the intensity profiles and dynamic event signals are asymmetric.

Using these characteristics, eye blinking in the entire video can be estimated by finding the value of “x_1_ ~ x_3_” and calculating “y_2_ − y_1_” and “y_2_ − y_3_”. If the estimated blink duration, “x_3_ − x_1_”, is shorter than 50ms or longer than 1500 ms, it is considered a non-blinking behavior, such as flutter or micro-sleep, and is excluded from the analysis process. Also, if the difference between the values ​​of “y_2_ − y_1_” and “y_2_ − y_3_” is large (> 20%), it is assumed that the signal change is due to a change in the environment, and is also excluded from the analysis. Then, all frames corresponding to each eye blink are extracted from the entire video, and each blink is saved as an individual video clip. Basic parameters such as frequency and duration can be calculated through this extraction task.

This process allows the calculation of blinking rate and duration which have been used as the main indicators of blink analysis in most previous studies on blink analysis regardless of the measurement method [9-13].

### Visualization of eye blinking pattern

The eye blinking rate and duration of eye blinking do not provide detailed information about the pattern of each eye blinking, but only indicate the overall tendency to eye blinking. For a more detailed analysis, this study aimed to precisely analyze eyelid movements during blinking by finding the position and shape of the eyelids in extracted eye blink video clips, to divide the blink sequence according to eyelid movement, and to classify the blink patterns. Furthermore a method was proposed to visualize the pattern of eye blinking as a single image so that the movement of the eyelids can be easily recognized.

#### Evaluation of the shape and position of the upper

**Fig. 2:**
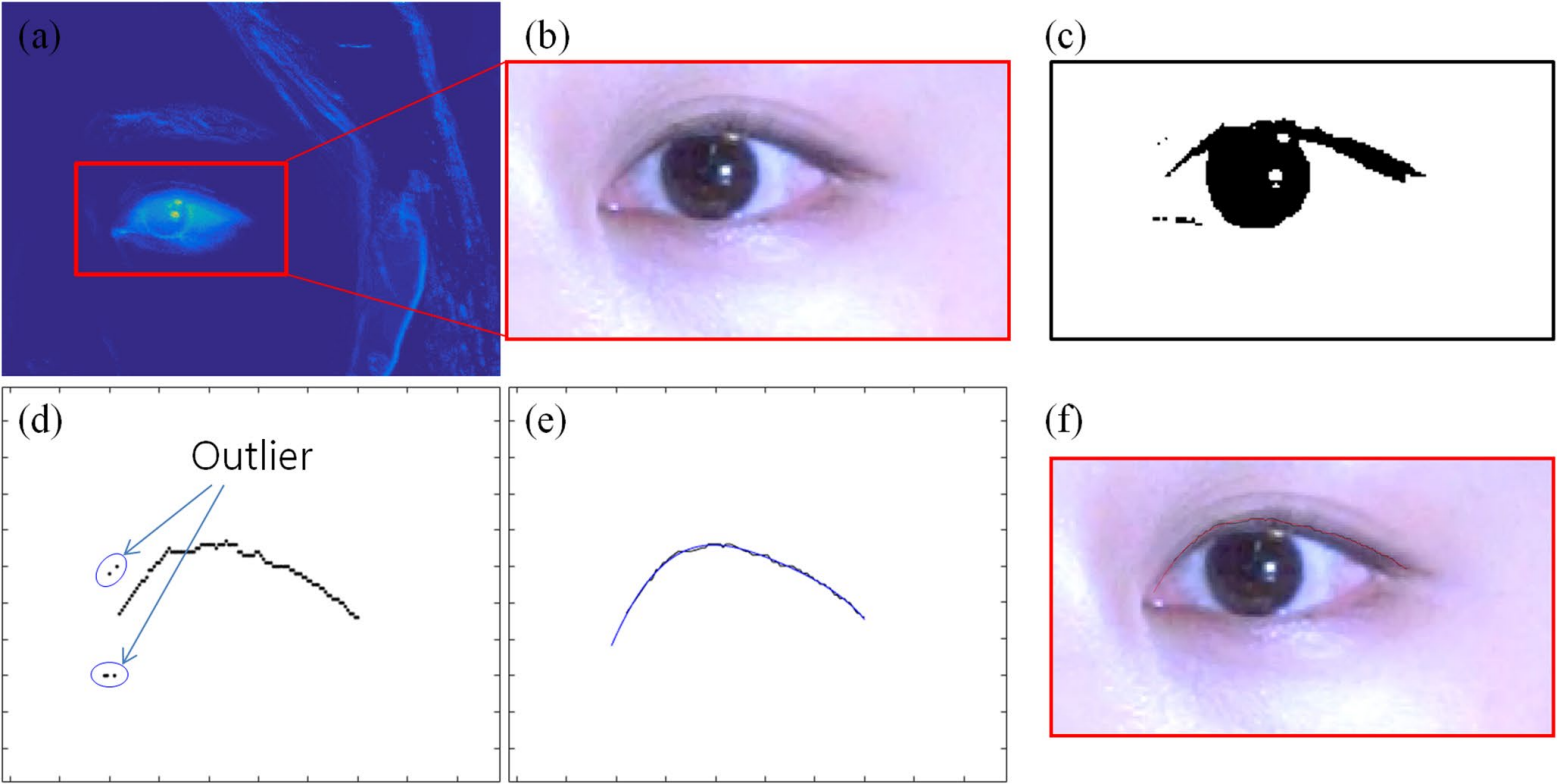
Process of evaluation of the shape and position of the upper eyelid. **a** The event data accumulation image during the eye blinking, **b** Cropped ROI image, **c** Binarization of (**b**), **d** Estimated upper edge with outlier, **e** Result of polynomial curve fitting without outlier, **f** Result image

 To visualize eye blinking patterns, the position and shape of the eyelid must be estimated from all frames during each blinking. To estimate the eyelid, the first procedure is to extract the region of interest (ROI) around the eye from the full frame. To achieve this, all the color frame images of the extracted eye blinking video clip are converted to binary images by a brightness threshold, which is the local minimum point in the histogram of the first frame image. Then, the differences between consecutive frames are calculated to generate differential images, and data from the generated differential images are accumulated into a single dynamic vision image, as shown in Fig. 2a. A dynamic visual image represents the frequency with which pixels change during a blinking, with brighter pixel indicating more frequent changes. The location of a rectangle surrounding the brightest blob in the dynamic image is designated as the ROI. The brightest blob represents the area that that changes most frequently during blinking, i.e., the eye.

From the image cropped at the ROI location in the full frame, the upper eyelid is roughly estimated by finding the upper black pixel of each column. The roughly estimated result may include outlier points that are not eyelids. Therefore, it is necessary to remove outlier points in order to obtain an accurate detection result.

An iterative outlier removal algorithm, which is a modified version of the Random Sample Consensus (RANSAC) algorithm, is used to remove outlier points. The RANSAC is an iterative method to estimate actual model parameters by removing outliers using repeated random sampling from a set of observed data that contains outliers [20, 21]. The basic RANSAC algorithm has the disadvantage of long computation time due to the iterative operations [22]. In order to reduce computation time, instead of performing random sampling from the beginning, we use a method of iteratively removing outlier points whose positions differ significantly from the adjacent pixels by applying a polynomial curve-fitting algorithm for every pixel.

After discarding outliers through iterative operations, the upper eyelid is estimated by interpolating the eyelid position of the empty column from the nearby pixels. Table 1 describes the removal algorithm [23] and the process and result of each step are shown in Fig. 2.

**Table 1:**
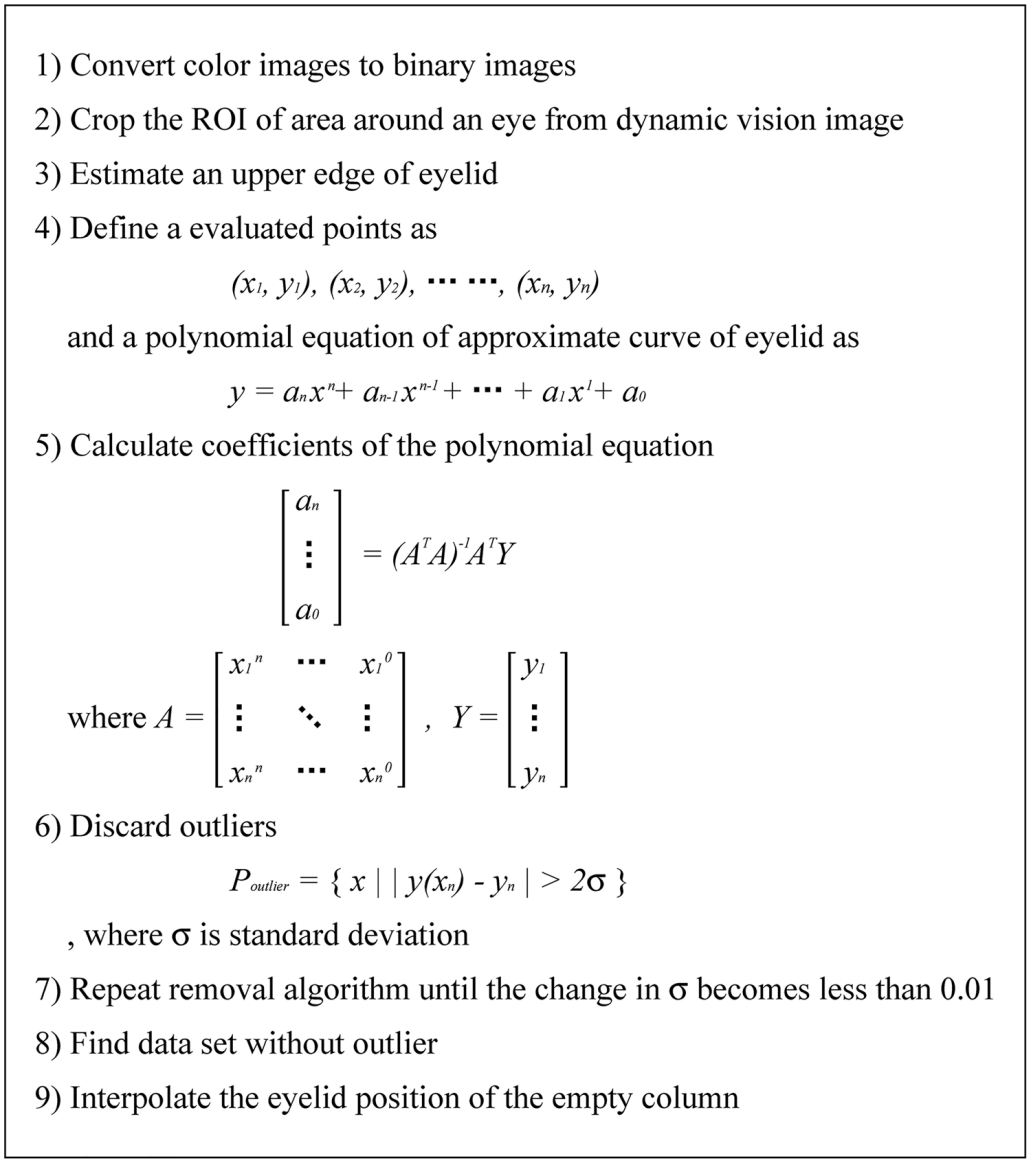
Iterative outlier removal algorithm for eyelid estimation

#### Visualization graph of eye blinking

It is very difficult to compare and analyze all the positions and shapes of the eyelids evaluated from each frame. Therefore, a method of recognizing all changes of the eyelids during eye blinking is necessary for easier and more detailed analysis of eye blinking patterns. In this study, a method for visualization of eye blinking patterns is proposed, which is to plot a 3-dimensional graph representing eyelid changes during the eye blinking and to make a single image by projecting the graph.

To generate a matrix for visualization, the horizontal (x-axis) domain size of evaluated eyelid data from each frame is made constant. At this time, the domain size is determined as the mode value of the pixel lengths of the eyelids estimated in each frame. If the estimated eyelid length in frame is longer than the domain length, some data on both sides are removed. Conversely, if the estimated eyelid length is shorter than the domain length, the empty data spaces are estimated using a polynomial curve equation. Then, the resized data are recorded in one matrix. For easy comparison between each eye blinking, the horizontal length of the all matrices is resized to 500 pixels, and the vertical value is normalized so that the y-axis displacement of the first frame is from 0.2 to 0.7.

**Fig. 3:**
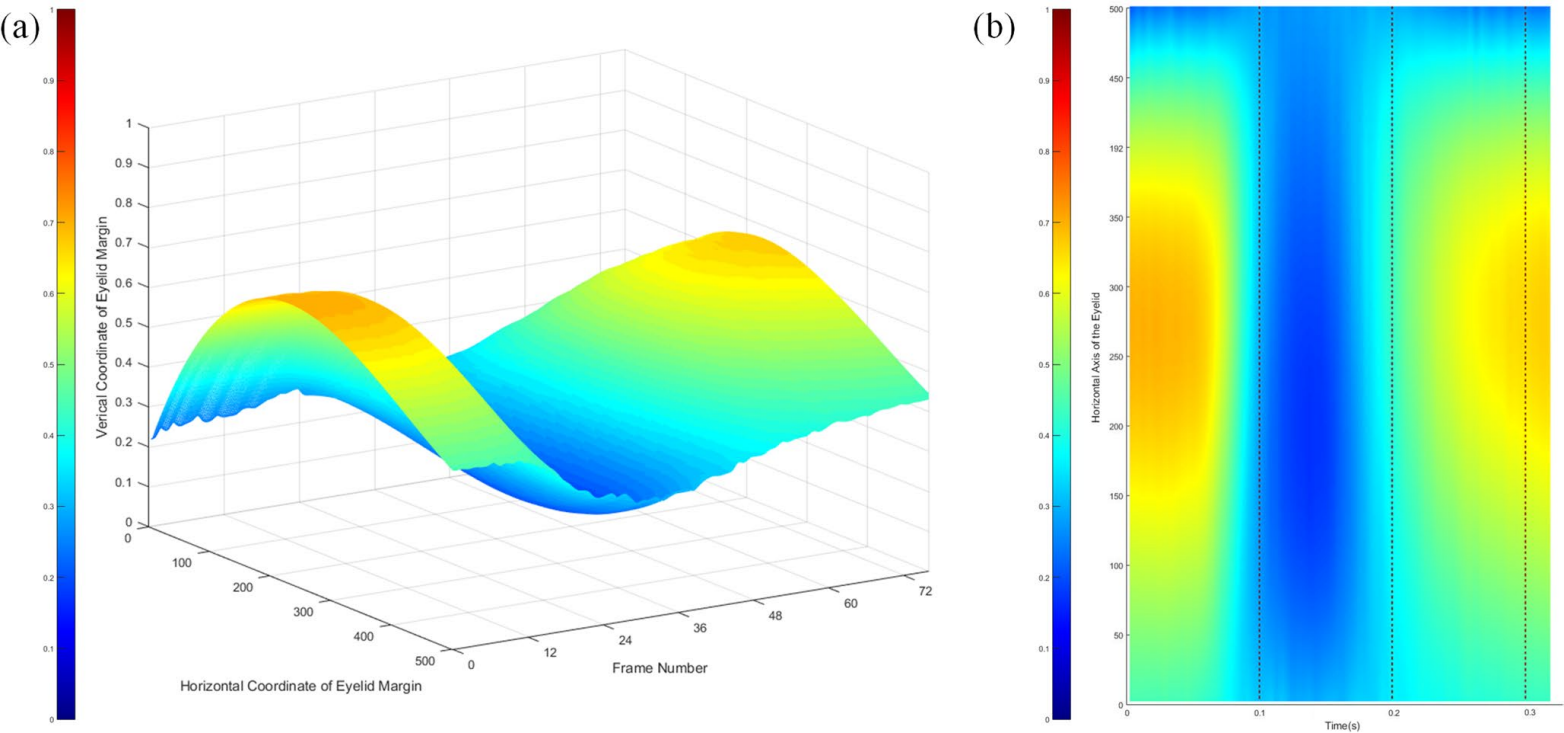
Visualization graph of an eye blinking **a** three-dimensional graph **b** projection image

The normalized matrix is plotted as a three-dimensional graph image represented using a color map of jet palette. The X-axis of the graph shows the change over time, and the YZ plane represents the shape of the upper eyelid. The visualization graph shows the changes in the shape of the evaluated upper eyelids over time during eye blinking. The eyelid changes during the blinking can be more easily recognized using a projection image onto a two-dimensional XY plane. Red dotted lines are added to the projection image at 0.1 s intervals to make it easier to compare the duration between different blinking images. The pattern of single eye blinking can be easily analyzed according to color changes of the graph (Fig. 3).

#### Eyelid displacement graph

The intensity profile shown in Fig. 1 does not accurately represent the displacement of eyelid movement. To accurately analyze eyelid movements, a displacement graph of the eyelid was generated by collecting the center position data extracted from the matrix created for the visualization. According to the change of the displacement graph, one eye blinking cycle is divided into three phases: ‘Closing phase’, ‘Complete Closed phase’, and ‘Opening phase’[24]. The ‘Complete Closed phase’ is defined as the section of corresponding to the lower 10% (less than 0.25) of the initial eyelid displacement. The front section of ‘Complete Closed phase’ is defined as ‘Closing phase’, and the subsequent section is defined as ‘Opening phase’. However, if the minimum displacement value of the eyelid position graph is greater than 0.3, the eye blinking is considered to have no ‘Complete Closed phase’. This case is defined as ‘Incomplete blinking’, which means that the eyes are not completely closed while blinking. The incomplete blinking has been cited as one of the reasons for dry eyes, so there have been studies to analyze it [13]. However, it has been very difficult to measure the eyelid position from the signal graph representing changes in bio-signal or pixel intensities, so it has been impossible to accurately define and analyze this blinking pattern.

**Fig. 4:**
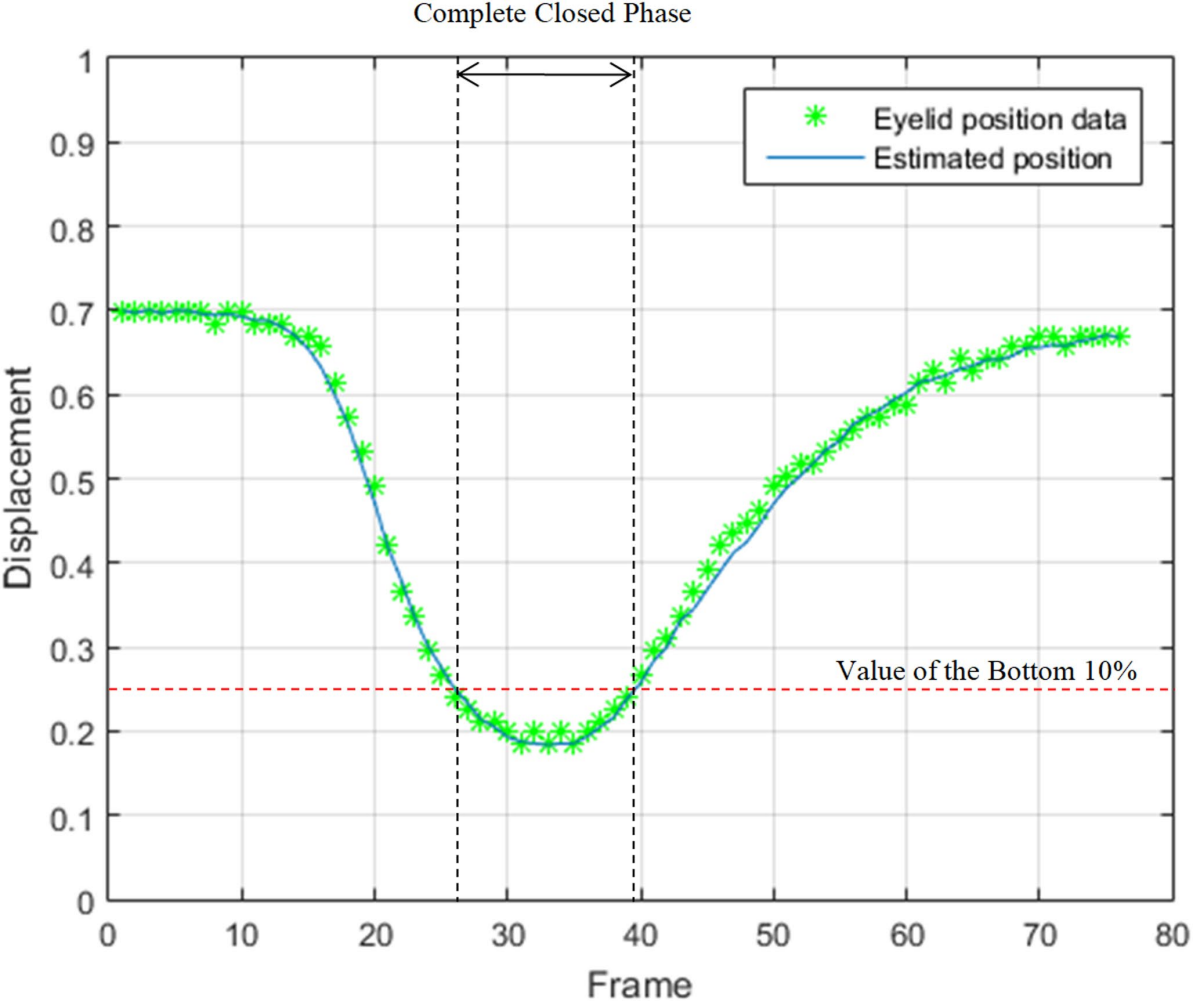
Displacement of eyelid position and selected ‘Complete Closed phase’

The various parameters regarding eye blinking can be calculated by the displacement graph; and calculable parameters included blinking duration, closed time, speed of closing and opening, ratio of complete closed phase, total displacement of upper eyelid (Fig. 4).

## Experimental result

An eye blink analysis experiment was conducted on 80 volunteers using the proposed method. To obtain various eye blinking videos, this study was conducted on 35 volunteers without any specific disease or symptoms, 25 volunteers with dry eye symptoms with tear breakup time (TBUT) of less than 7 s, and 20 volunteers diagnosed with Parkinson’s disease. The video used for analysis was taken while volunteers watched television for five minutes from a distance of 1.1 m.

We calculated eye-blinking-related parameters, which were primarily used in studies analyzing eye blinking using bio-signals, based on recorded high-frame-rate videos of volunteers. Table 2 shows the result of calculating the eye blinking-related parameters. The volunteers blinked an average of 16.12 times per minute, and the average blinking duration was 284.5ms. This is not significantly different from the average blinking rate (15 ~ 20 times per minute) and the average blinking duration (100 ~ 400 ms) reported in other previous experiments [25]. We were also able to detect ‘incomplete blinking’ that cannot be detected by bio-signal analysis.

**Table 2:** Parameters of experimental result with 80 volunteers

Parameter	Value			
	Normal	Dry eye	Parkinson	Total
Mean of Blinking rate (Blink / Minute)	17.52	20.55	8.22	16.12
Mean of Blinking Duration (milliseconds)	291.8	293.7	218.6	284.5
Mean of standard deviation of Individual Blinking Duration	49.21	84.64	52.85	61.63
Proportion of Closure Phase (%) (sum of blinking time / total time)	8.53	9.08	2.99	7.32
Ratio of Incomplete blinking (%)	4.87	9.03	14.29	7.39

**Fig. 5:**
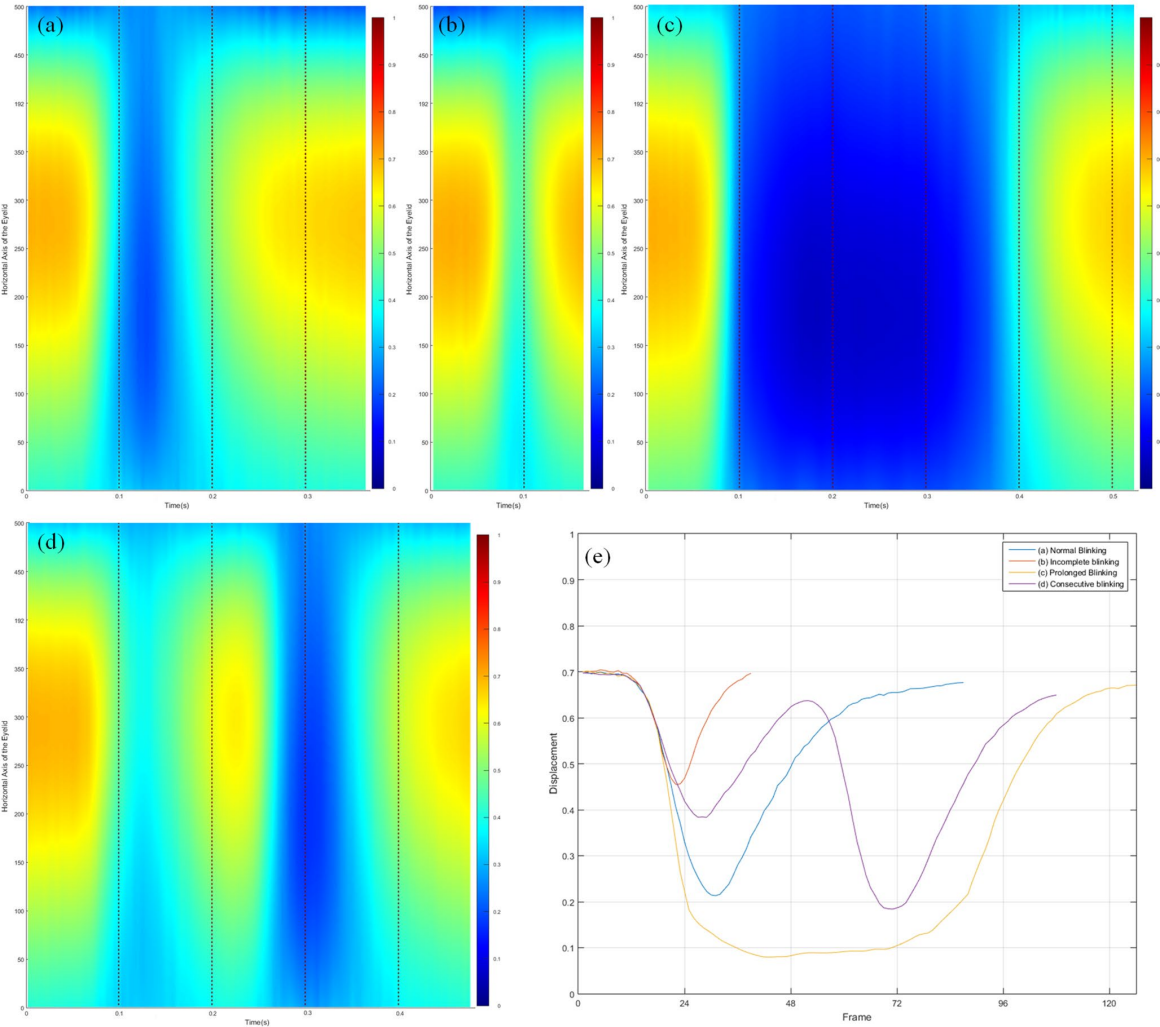
Visualization results for various eye blinks. **a** Normal blinking pattern, **b** Incomplete blinking, **c** Prolonged Blinking, **d** Consecutive blinking, **e** Eyelid position graph of (**a**) ~ (**d**)

Additionally, we further analyzed the eye blinking patterns using visualization images and eyelid position graphs. Analysis of visualization results revealed four different patterns among the eye blinkings. Examples for each case are shown in Fig. 5, which are visualization results of eye blinks of different patterns.

The Fig. 5a shows a typical eye blinking pattern. The total duration of the eye blinking was 362 ms, and the duration of each phase was 116 ms, 30 ms, and 216 ms. That is, the duration when the eyes were judged to be completely closed was approximately 8.3% of the entire sequence. It took longer for the eyes to open than it did for them to close, although the eyelids moved the same distance. This indicates that the eyelids close faster than they open.

The visualization result shown in Fig. 5b was the incomplete eye blink defined in 2.3.3. For this incomplete blink, the blink duration was as short as 162.5 ms, which was about half of the average blink duration (291.8 ms) shown in Table 2. Additionally, the minimum value of the middle eyelid position was 0.456, which means that the eyes moved 0.244 and were only about half closed.

Conversely, Fig. 5c shows a prolonged blinking with a very long duration. The total duration of the eye blinking was over 525ms, and the duration of each phase was 125ms, 208ms, and 192ms. The times of ‘closing phase’ and ‘Opening phase’ did not differ significantly compared to normal eye blinking in Fig. 5a, but ‘Complete Closed phase’ was significantly longer, accounting for approximately 40% of the entire sequence.

This trend can also be confirmed in Fig. 5e. Although the duration varied, the speed at which the eyelids closed or rose was relatively constant. For this reason, it can be considered that blinking for a short duration appears as an incomplete blink because the eyelids open before they fully cover the eye. Conversely, longer blinking times were associated with an increase in the complete closed phase, i.e., the duration that the eyes remain completely closed.

Figure 5d is a visualization result of an abnormal eye blinking pattern, in which two eye blinkings occur consecutively. In this pattern, the first blinking occurred incompletely, the second blinking was initiated before the eyelids had fully opened in the first blinking. Because changes in eyelid position could be quantitatively measured, it was possible to distinguish between two normal blinks occurring within a short interval. In this case, we defined it as the ‘Consecutive Blinking’. Consecutive blinking is actually a common phenomenon and has been reported in many previous studies as a pattern that is highly related to drowsiness [26]. 

## Discussion

In this study, we proposed a method to analyze and visualize eye blink patterns using high-frame-rate videos. The experimental setup for analysis required only high-speed camera without additional equipment, and measurement were performed by a non-contact manner. These characteristics can overcome the limitations in the application and use of conventional bio-signal analysis methods that are only used in restricted places because electrodes and wires are always required.

The proposed method not only enabled the calculation of the various parameters utilized in other studies with bio-signal analysis, but also made the analysis of eye blinking patterns easy and natural, represented as graph images. In addition, the new method made it possible to quantitatively analyze the position of the eyelid, which is difficult to estimate using previous analysis methods. According to the change of the eyelid displacement graph, which is created by center position data extracted from the visualization matrix, an eye blinking cycle was divided into three phases: ‘Closing phase’, ‘Complete Closed phase’, and ‘Opening phase’. Then, the eye blinking without the ‘Complete Closed phase’ could be defined as an ‘Incomplete blinking’. Furthermore, it was possible to distinguish whether two normal blinks occurred within a short interval or whether the second blinkbegan before the first blink ended. The latter case is defined as a ‘Consecutive Blinking’.

Eye blink analysis was possible regardless of head movement, accessories worn, or illumination changes. However, the new analysis using the proposed method has also limitations. If eyes are outside the video frame or are covered by glasses, hair, etc., the changes in eyes and eyelids cannot be completely estimated. Nevertheless, quantitative data on eyelid changes obtained from video frame images are expected to be readily applicable to a wider range of fields because they contain more information than data obtained from bio-signals.

 Our proposed analysis could potentially be applied to in-cabin driver monitoring systems (DMS) in the automotive industry. With the recent enactment of laws regarding driver monitoring systems, research on driver monitoring systems is actively underway. The proposed analysis has the same equipment settings as the general DMS previously studied, so it can be applied without additional settings. By adding this analysis method, it might be possible to analyze not only the driver’s movements but also the driver’s condition. For similar reasons, it could be applied to head mounted display (HMD) and smart glasses for extended reality (XR) and could be used to prevent computer vision syndrome in office workers [19]. Since the user of these applications do not move away from the fixed camera, the limitations of proposed analysis methods can be somewhat mitigated.
